# The comparison of dexamethasone and triamcinolone periarticular administration in total knee arthroplasty: retrospective cohort study

**DOI:** 10.1186/s12891-022-05048-8

**Published:** 2022-02-05

**Authors:** Atsufumi Oshima, Kazuhisa Hatayama, Masanori Terauchi, Hibiki Kakiage, Shogo Hashimoto, Hirotaka Chikuda

**Affiliations:** 1 Department of Orthopaedic Surgery, Japan Community Health Care Organization Gunma Central Hospital, 1-7-13 Koun-cho, Maebashi, Gunma 371-0025 Japan; 2 Department of Orthopaedic Surgery, Gunma University Graduate School of Medicine, 3-39-15 Showa-machi, Maebashi, Gunma 371-8511 Japan

**Keywords:** Total knee arthroplasty, Periarticular injection, Corticosteroid, Dexamethasone, Triamcinolone acetonide

## Abstract

**Background:**

Intraoperative periarticular injection of corticosteroid effectively reduces perioperative pain in total knee arthroplasty (TKA). However, which corticosteroid is most effective for intraoperative periarticular injection remains controversial. We compared the effects of corticosteroids between dexamethasone and triamcinolone acetonide periarticular administration for reducing pain and postoperative nausea and increasing fasting blood glucose concentrations during the perioperative period following TKA.

**Methods:**

One hundred and two patients who underwent TKA from August 2018 to September 2020 were divided into two groups: one received 10 mg dexamethasone for intraoperative periarticular injection and another receiving 40 mg triamcinolone acetonide. Postoperative pain scores at rest and during walking and nausea scores were recorded using a 0-to-10 Numerical Rating Scale. C-reactive protein (CRP) and fasting blood glucose levels were measured pre- and postoperatively.

**Results:**

Pain scores in the triamcinolone group were significantly lower than in the dexamethasone group at rest 7 days postoperatively (1.5 vs. 2.0; *p* = 0.046) and while walking at both 72 h (3.9 vs. 4.8; *p* = 0.008) and 7 days postoperatively (3.2 vs. 4.0; *p* = 0.03). The CRP levels in the triamcinolone group were significantly lower than in the dexamethasone group at 7 days postoperatively (1.6 mg/dl vs. 3.0 mg/dl: *p* < 0.001). The fasting blood glucose levels at 1 day postoperatively were increased in both groups but not significantly different between the groups. No significant differences in the nausea score were noted between the groups.

**Conclusions:**

Triamcinolone acetonide periarticular administration provided greater pain relief by reducing inflammation to a greater degree than dexamethasone.

## Background

Intraoperative administration of corticosteroid during total knee arthroplasty (TKA) has been reported to reduce perioperative pain and prevent nausea [1–8], and the routes of administration include intravenous and periarticular administration.

A few studies thus far have compared the effects of intravenous and periarticular corticosteroid administration during TKA. These studies suggested that periarticular administration was recommended over intravenous administration for postoperative pain relief [9, 10]. However, no studies have yet determined which steroids are most effective with periarticular administration during TKA. For example, in rheumatoid arthritis, intra-articular injection of 8 mg dexamethasone and 40 mg triamcinolone acetonide was reported to reduce knee joint pain and swelling significantly, but no significant differences in knee pain between the two groups were noted [11].

In addition, referring to the concern of hyperglycemia caused by the administration of corticosteroids, a previous study showed that the fasting blood glucose levels of the patients treated with triamcinolone acetonide periarticular injections were significantly increased the morning after TKA [9]. Several other studies have further reported that the intravenous administration of dexamethasone did not increase fasting blood glucose levels postoperatively following TKA [1, 12]. However, no studies have yet described the effects of periarticular injections of dexamethasone on blood glucose levels after TKA.

Given the above, the present study compared the corticosteroid effects of periarticular injection between dexamethasone and triamcinolone acetonide on reducing pain and postoperative nausea and increasing fasting blood glucose levels during the perioperative period in TKA. We hypothesized that triamcinolone acetonide would provide greater pain relief by reducing inflammation to a greater degree than dexamethasone.

## Methods

After receiving approval from the institutional review board, we retrospectively identified two cohorts of patients who had undergone unilateral TKA from August 2018 to October 2020. The triamcinolone group included 50 patients from the previous randomized controlled study [9]. After completion of the study, we added 52 patients to the dexamethasone group based on the same criteria. The inclusion criteria were (1) primary unilateral TKA for osteoarthritis of the knee and (2) age 55 to 90 years old. The exclusion criteria were patients with (1) a history of contralateral TKA within one year, (2) the administration of any glucocorticoids during the three months prior to the surgical procedure, (3) diabetes mellitus, (4) rheumatoid arthritis, (5) a history of knee surgery or knee injury for example high tibial osteotomy, meniscus repair, ligament reconstruction and so on (6) liver or renal failure (7) administration of any anticoagulant drug, and (8) preoperative deep venous thrombosis (DVT).

In the dexamethasone group, at the period from May 2020 to October 2020, a periarticular injection of 60 mL of physiological saline solution containing 10 mg dexamethasone, which has been confirmed to be an effective dose, [5] and 150 mg ropivacaine was administered during TKA.

In the triamcinolone group, at the period from August 2018 to May 2020, a periarticular injection of 60 mL of physiological saline solution containing 40 mg triamcinolone acetonide, which has been confirmed to be an effective dose, [13] and 150 mg ropivacaine was administered. The biological half-lives of dexamethasone and triamcinolone acetonide are 36 to 55 h and 18 to 36 h, respectively, and 40 mg of triamcinolone has an anti-inflammatory potency equivalent to 8 mg of dexamethasone [2, 14, 15].

All TKA procedures were performed under general anesthesia. A single-shot, echo-guided femoral nerve block consisting of 20 mL of physical saline solution containing 75 mg ropivacaine was performed for all patients before the surgical procedure. Two senior surgeons performed or assisted in all surgical procedures. A pneumatic tourniquet was inflated to 250 mmHg. Medial parapatellar arthrotomy was performed. An Attune prosthesis (Depuy, Warsaw, IN, USA) was implanted in all knees using cement. Just before the prosthesis was implanted, 20 mL of the mixture was injected into the posterior aspect of the capsule and the remaining 40 mL was injected into the synovium, periosteum, iliotibial band, and collateral ligaments. After capsule closure, all patients were administered an intra-articular injection of 2 g tranexamic acid in 20 mL of physiological saline solution to reduce blood loss. The tourniquet was released after the wound had been closed and the knee bandage had been wrapped. No drain was inserted in any cases.

Pain and nausea control were performed similarly in all patients. From the day after surgery, 1 g acetaminophen was taken orally 3 times a day. For the management of pain, an intramuscular injection of 15 mg pentazocine and transrectal administration of 25 mg diclofenac were given if the patient required rescue. For the management of nausea, an intramuscular injection of 10 mg metoclopramide was given if the patient requested it. All patients were allowed to walk with a walker on the day after surgery. The number of uses of rescue analgesia and metoclopramide was recorded for 24 h postoperatively. Pain scores at rest, pain scores during walking, and nausea scores were recorded using a 0-to-10 Numerical Rating Scale, with pain at rest recorded at 4, 8, 16, 24, 48, and 72 h and 7 days postoperatively; pain during walking at 24, 48, and 72 h and 7 days postoperatively; and nausea at 8, 24 and 48 h postoperatively. The number of patients who vomited within 24 h after surgery was recorded. Patient temperatures were measured every 6 h for 72 h after surgery, and patients who had a high fever over 38 °C at any point were recorded.

Demographic information, including the age, sex, body weight, height and operative time, were recorded. CRP and fasting blood glucose levels were determined within three weeks preoperatively. Fasting blood glucose levels were determined the morning after surgery. CRP levels were determined 24 and 48 h and 7 days postoperatively.

**Fig. 1:**
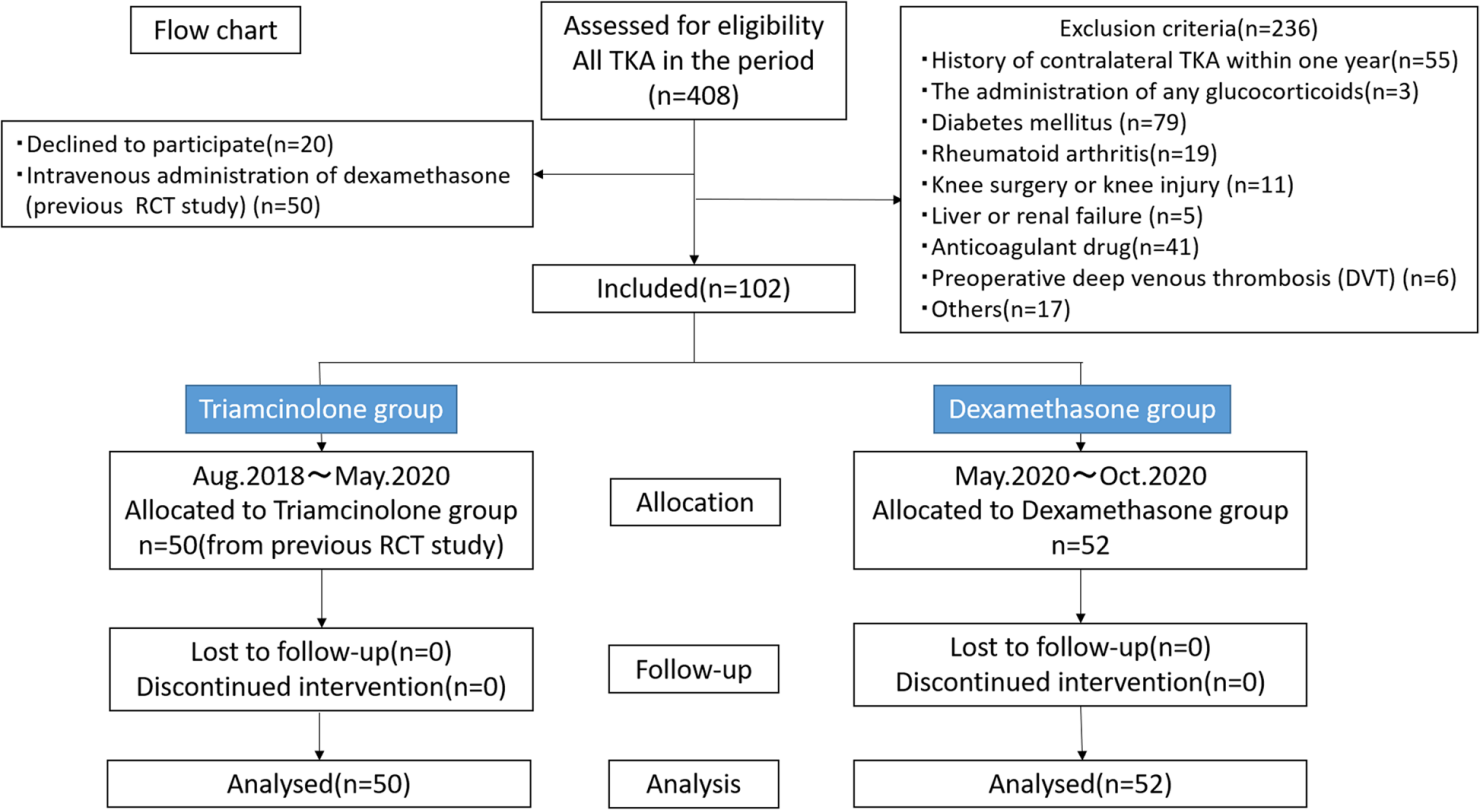
Flow chart

Based on the results of previous studies, [2, 16] an a priori power analysis was performed to detect a difference in pain score of 0.8 with the desired power of 0.90 and a significance level of 0.05. The required sample size was calculated as 40 for each arm of the study. Assuming a 10% exclusion rate, the minimum sample size was 44 in each group. We included over 50 patients in each group (Fig. 1).

All statistical analyses were performed using the SPSS software program (SPSS version 26.0; IBM, International Business Machines Corporation, Armonk, NY, USA). Student’s *t*-test was used to compare the age, body weight, height, operative time, CRP level, and fasting blood glucose level between the groups. The chi-square test was used to compare the sex, incidence of a postoperative fever, and incidence of vomiting between the groups. The Mann-Whitney U-test was used to compare the pain and nausea scores and number of times rescue analgesia and metoclopramide were used between the groups. For all parameters other than the pain score, a post hoc analysis was performed to confirm the power of the test with significant differences.

## Result

Fifty-two patients were allocated to the dexamethasone group, and 50 patients were allocated to the triamcinolone group (Fig. 1). Preoperative fasting blood glucose levels were significantly higher in the triamcinolone group than in the dexamethasone group, but other demographic characteristics and preoperative laboratory data showed no significant differences between the groups (Table 1).

**Table 1:** Patient demographics and preoperative laboratory data

	Dexamethasone	Triamcinolone	*p* value
Age (year)	74.1 ± 7.6	72.0 ± 6.2	0.133
Sex (M/F)	9/43	12/38	0.468
Height (cm)	151.4 ± 7.9	154.0 ± 9.2	0.120
Weight (kg)	59.2 ± 10.6	61.4 ± 9.7	0.279
Operative time (min)	100 ± 16	101 ± 15	0.676
FBG (mg/dL)	102.9 ± 9.2	107.7 ± 14.0	0.041
CRP (mg/dL)	0.20 ± 0.44	0.24 ± 0.57	0.686
flex-active ROM (°)	122.8 ± 15.4	122.5 ± 12.7	0.902

*FBG* Fasting Blood Glucose

*ROM* Range of Motion

Values are the mean ± standard deviation

**Fig. 2:**
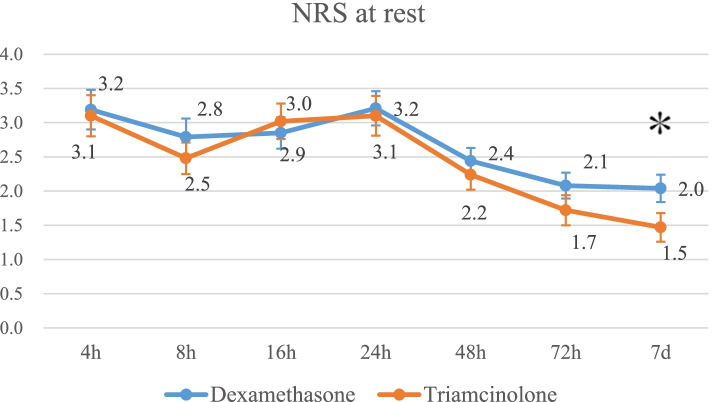
Mean pain score at rest for each group. The error bars indicate the standard error of the mean. *p < 0.05

**Fig. 3:**
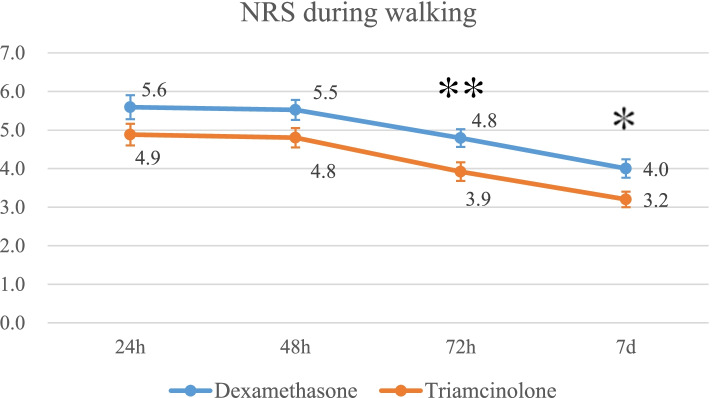
Mean pain score during walking for each group. The error bars indicate the standard error of the mean. **p < 0.01 *p < 0.05

The pain scores in triamcinolone group were significantly lower than those in the dexamethasone group at rest at 7 days after surgery (mean and standard deviation, 1.5 ± 1.5 vs. 2.0 ± 1.5; *p* = 0.046) (Fig. 2) and while walking both at 72 h (3.9 ± 1.7 vs. 4.8 ± 1.7; *p* = 0.008) and at 7 days after surgery. (3.2 ± 1.4 vs. 4.0 ± 1.7; *p* = 0.03) (Fig. 3) There were no significant differences in the number of uses of rescue analgesia between the two groups (Table 2).

**Table 2:** The number of use of rescue analgesia and metoclopramide and the incidence of fever and vomiting

	Dexamethasone	Triamcinolone	p value
Mean number of the uses of rescue analgesia	1.3 ± 0.8	1.1 ± 0.8	0.330
Number of patients who had a fever	4 (7.7%)	2 (4%)	0.551
Mean number of the uses of metoclopramide	0.3 ± 0.5	0.3 ± 0.5	0.325
Number of patients who had vomiting	5 (9.6%)	7 (14%)	0.678

Values are the mean ± standard deviation

**Fig. 4:**
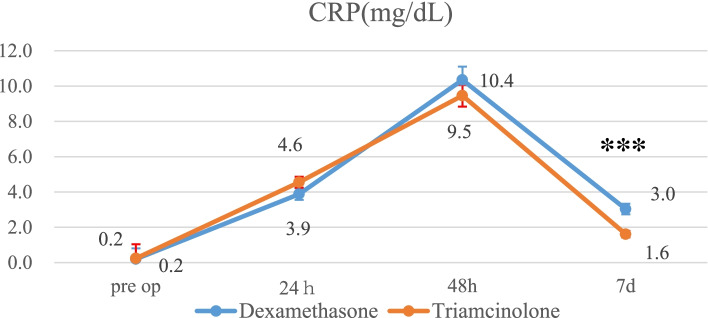
Mean CRP for each group. The error bars indicate the standard error of the mean. ***P < 0.001

CRP in the triamcinolone group is significantly lower than in the dexamethasone group at 7 days after surgery. (1.6 ± 1.3 mg/dl vs. 3.0 ± 2.2 mg/dl: *p* < 0.001: post hoc power 0.98) (Fig. 4). Four patients (7.7%) had a fever in the dexamethasone group, whereas 2 (4%) had a fever in the triamcinolone group. There were no significant differences in the number of patients who had a fever within 72 h postoperatively between the two groups (Table 2).

**Fig. 5:**
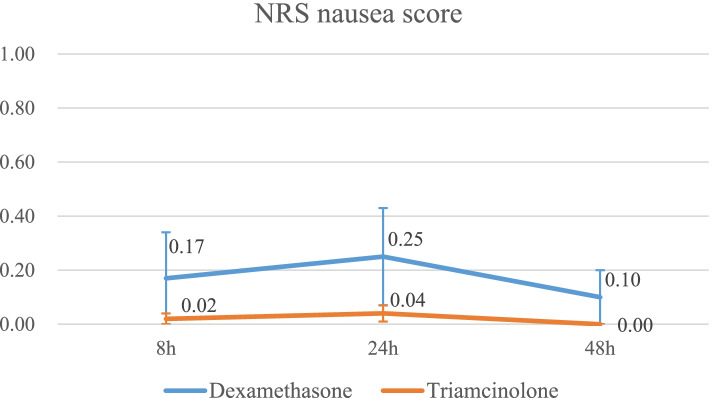
Mean nausea score for each group. The error bars indicate the standard error of the mean

There were no significant differences in the nausea score between the two groups (Fig. 5). Five patients (9.6%) vomited in the dexamethasone group, and 7 patients (14%) vomited in the triamcinolone group, showing no significant differences. There were also no marked differences in the incidence of metoclopramide use between the two groups. (Table 2).

**Fig. 6:**
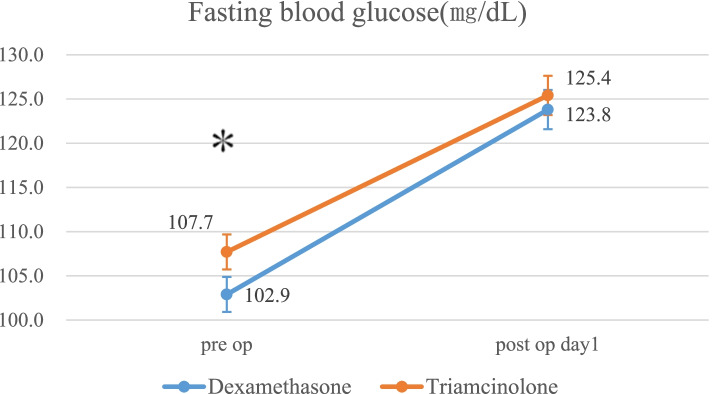
Mean fasting blood glucose for each group. The error bars indicate the standard error of the mean. *p < 0.05

Fasting blood glucose levels the morning after surgery were significantly increased in both groups compared with before surgery (dexamethasone group: preoperatively 102.9 ± 9.2 mg/dl → postoperatively 123.8 ± 14.3 mg/dl; triamcinolone group: preoperatively 107.7 ± 14.0 mg/dl → postoperatively 125.4 ± 15.7 mg/dl: post hoc power 0.53). However, there were no significant differences in the levels between the two groups after surgery (Fig. 6).

## Discussion

The most important finding in this study was that the pain scores in the triamcinolone group were significantly lower than those in the dexamethasone group while walking at 72 h and both at rest and while walking at 7 days after surgery. In addition, the CRP levels in the triamcinolone group were significantly lower than those in the dexamethasone group at 7 days after surgery.

Periarticular administration of corticosteroids proved effective for reducing perioperative pain for TKA [1–3, 14–16]. Many different types of corticosteroids have been used for periarticular injection [17]. However, which is the most effective type of corticosteroid is unclear. To our knowledge, this study is the first to compare the effects of different types of corticosteroids delivered via periarticular administration following TKA.

Previous pharmacokinetic studies have shown that 8 mg of dexamethasone is equivalent to 40 mg of triamcinolone [2, 14, 15]. Dexamethasone is insoluble in water, has an average intraarticular duration of action of 8 days, and has a clinical dose of 8 mg [18].. In contrast, triamcinolone acetonide is insoluble in water, has a clinical dose of 10-40 mg, and has a longer duration of action of 14 days [18–20]. In the present study, 40 mg triamcinolone acetonide periarticular administration provided greater pain relief and more markedly reduced inflammation than 10 mg dexamethasone, with a particular difference seen in the results from 72 h to 7 days. In addition, the CRP levels in the triamcinolone group were significantly lower than in the dexamethasone group at 7 days after surgery. Our findings showed that triamcinolone acetonide had a stronger long-term analgesic and anti-inflammatory effect than dexamethasone in a clinical setting.

A postoperative fever after TKA is partly the result of the local and systemic release of inflammatory cytokines [21]. A previous study showed that 10 of 20 patients (50%) had a fever of > 38.5 °C for 3 days after TKA without glucocorticoid administration [21]. Another systematic review reported that 62 of 170 patients (36.5%) had pyrexia (> 38.0 °C) in the first 5 days following TKA without glucocorticoid administration [22]. In the present study, 4 of 52 (7.7%) patients who were provided dexamethasone and 2 of 50 (4%) who were provided triamcinolone acetonide had a fever (> 38.0 °C) within 72 h after TKA. These rates were lower than those reported previously in studies without corticosteroid administration. Both dexamethasone and triamcinolone acetonide periarticular injections thus appear to have had anti-inflammatory effects, and their effects were equivalent in the short term.

Another benefit of the administration of corticosteroids is the postoperative antiemetic effect. Most postoperative nausea occurs during the first 24 h after surgery, and the intravenous administration of dexamethasone effectively prevents postoperative nausea [2, 3]. Periarticular administration also had a postoperative antiemetic effect [23]. Another study showed that periarticular injection of corticosteroids had a similar antiemetic effect to intravenous administration [9]. Our present study showed that the periarticular injection of corticosteroids reduced postoperative nausea and vomiting regardless of the type of corticosteroid, at least for dexamethasone and triamcinolone acetonide.

The effect of corticosteroids on postoperative hyperglycemia after TKA is controversial at present. Hyperglycemia of > 200 mg/dL increased the risk of superficial surgical site infection and wound complications [24, 25]. A previous study reported that there was no association between perioperative dexamethasone intravenous administration during arthroplasty and the risk of maximum postoperative hyperglycemia > 200 mg/dl [26].. However, in another previous study, the fasting blood glucose level on the morning following surgery in patients who received periarticular injection of triamcinolone acetonide was significantly higher than that in patients who received intravenous administration of dexamethasone [9]. In the present study, the fasting blood glucose level the morning following surgery was increased in both groups who received periarticular injection of triamcinolone or dexamethasone, although no patients had hyperglycemia > 200 mg/dL. When administering periarticular injections, regardless of the type of corticosteroid, we should be alert for increased blood glucose levels.

Several limitations associated with the present study warrant mention. First, the period during which the two corticosteroids were selected was different. The lack of a control group in the same study period may over-estimate the results [27]. Second, a femoral nerve block was performed for all patients before TKA, which may have masked the pain scale in early the postoperative period. The analgesic effects of reduced pain due to a peripheral nerve block and periarticular injection have been reported to be similar for TKA [28]. Third, the pain scores in the triamcinolone group were significantly lower than those in the dexamethasone group, ranging from 0.4 to 0.9 at the NRS. However, the minimal clinically important difference (MCID) for the visual analog scale for pain was 16.1 mm for TKA with minimal detectable change [29]. These significant differences in pain score may not be clinically important differences.

## Conclusions

Triamcinolone acetonide periarticular administration provided greater pain relief by more effectively reducing inflammation than dexamethasone. There were no significant differences in the control of the fasting blood glucose level or nausea between triamcinolone acetonide and dexamethasone.

## Data Availability

The datasets used and/or analysed during the current study are available from the corresponding author on reasonable request.

## Abbreviations

TKA: Total knee arthroplasty
CRP: C-reactive protein
MCID: The minimal clinically important difference
